# Supplementary material for: KSHV 3.0: a state-of-the-art annotation of the Kaposi’s sarcoma-associated herpesvirus transcriptome using cross-platform sequencing
Source: mSystems. 2024 Jan 11;9(2):e01007-23. doi: 10.1128/msystems.01007-23 (PMC10878076; doi:10.1128/msystems.01007-23)
Supplement: Figure S1 — Transcriptional end sites of KSHV RNAs. [file msystems.01007-23-s0001.pdf]

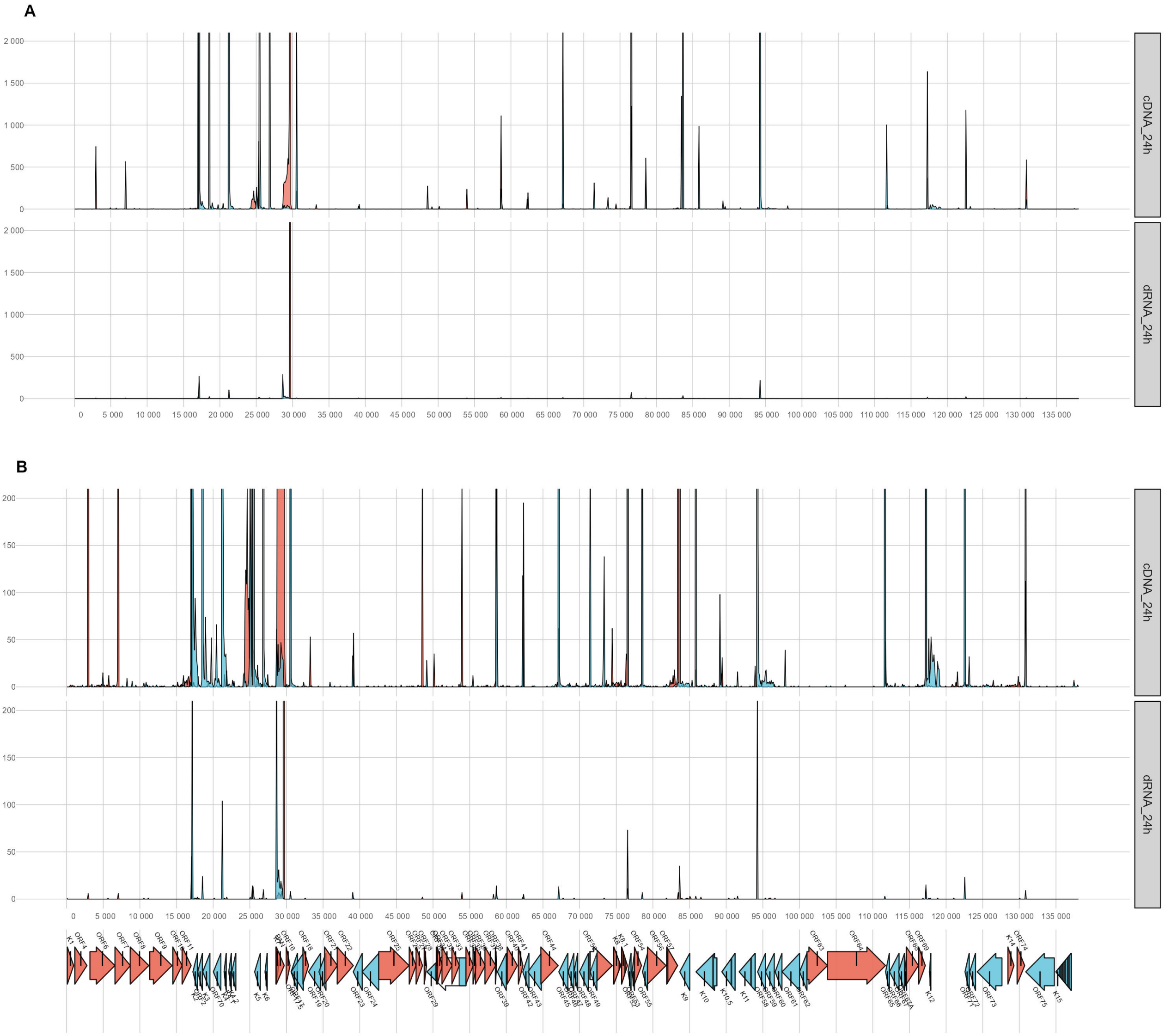

Supplemental Figure 1. Transcriptional end sites of KSHV RNAs

In this figure, the TES distribution aligned with the KSHV genome annotation is illustrated, highlighting each ORF. Panels A and B illustrate the coverage from the dcDNA and dRNA sequencing datasets derived from the 24 hpi samples, respectively. For each nucleotide the TES signal strength value was calculated by counting the reads that have their 3' ends aligning with that specific position. In the context of the dcDNA-Seq, reads were only included if their orientation could be identified through the presence of either 5' or 3' adapters. The dcDNA-Seq data of the three replicates were merged. Subsequently, the TES signal strength values were clustered in 100-nt intervals to represent the distributions. The y-axis in panel (A) tops out at 2000 reads, whereas in panel (B) it is limited to 200 reads. Gene orientations are color-coded: red for the positive strand and blue for the negative strand.
