# Supplementary material for: KSHV 3.0: a state-of-the-art annotation of the Kaposi’s sarcoma-associated herpesvirus transcriptome using cross-platform sequencing
Source: mSystems. 2024 Jan 11;9(2):e01007-23. doi: 10.1128/msystems.01007-23 (PMC10878076; doi:10.1128/msystems.01007-23)
Supplement: Figure S2 — Introns, TSSs, and TESs of KSHV transcripts. [file msystems.01007-23-s0002.pdf]

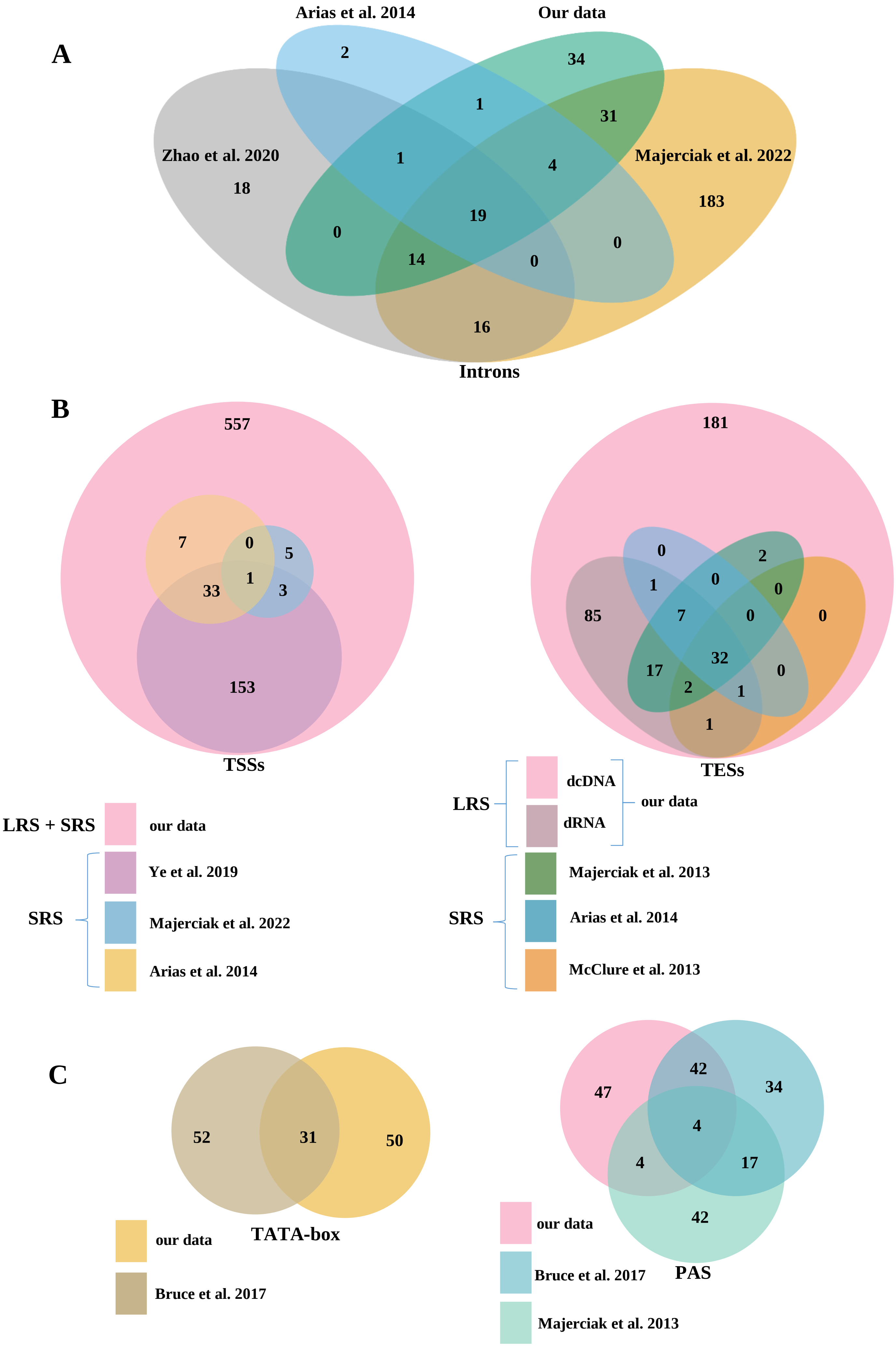

Supplemental Figure 2. Introns, TSSs and TESs of KSHV transcripts

A. The Venn-diagram displays the count of introns sourced from published data and compares them with our findings obtained through dRNA and dcDNA sequencing.

B. The TSS diagram illustrates the cumulative count of annotated 5′ read ends utilized in the transcript assembly as TSS. These positions were compared to previously annotated TSSs and the diagram shows the overlap between them entitled by the publication sources. The TES diagram shows the number of annotated 3′ read ends utilized in the transcript annotation as TES. The oligo(dT)-based dcDNA-Seq and dRNA-Seq yield comparable 3′ ends for mRNAs. Our TES positions were compared to previously annotated TESs and the diagram shows the overlap between them entitled by the publication sources. End positions of reads were counted within a +/-10 bp window.

C. The Venn-diagrams show the overlap between the previously known and novel TATA boxes, as well as the overlap between the newly detected and already annotated poly(A) signals.
