# Supplementary material for: KSHV 3.0: a state-of-the-art annotation of the Kaposi’s sarcoma-associated herpesvirus transcriptome using cross-platform sequencing
Source: mSystems. 2024 Jan 11;9(2):e01007-23. doi: 10.1128/msystems.01007-23 (PMC10878076; doi:10.1128/msystems.01007-23)
Supplement: Figure S3 — Heterogeneity of transcription initiation and termination. [file msystems.01007-23-s0003.pdf]

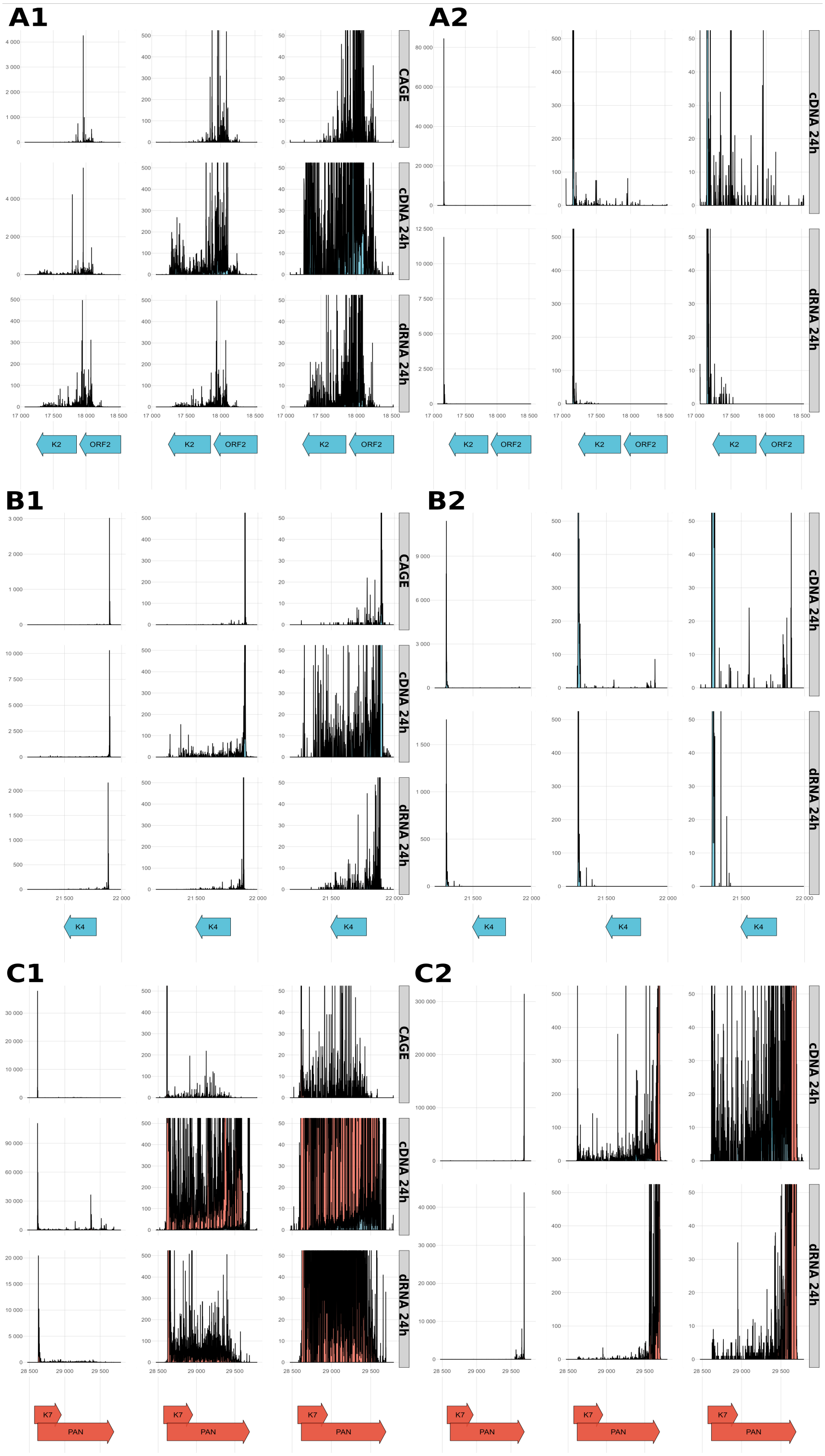

Supplemental Figure 3. Heterogeneity of transcription initiation and termination

The figure is composed of six panels, illustrating a wide variety of TSS (A1, B1 and C1) and TES (A2, B2 and C2) distributions along three genomic regions. The upper panel (A1-A2) shows the K2-ORF2 genomic region, the middle panel shows the region around K4 (B1-B2), while the bottom panel (C1-C2) shows the K7-PAN region. The x-axes show the genomic position, while the y-axes represent the number of either 5-prime ends, or 3-prime ends of reads in that position for the TSS or TES panels, respectively. For each panel, there are three sub-panels in different scales: the leftmost panels have no y-axis limit, the middle panels have a limit of 500 reads, while the rightmost panels have 50 reads. Genes depicted on the figure show examples for both TSS and TES clustering, which phenomenon is independent from the sequencing method.
