# Supplementary material for: KSHV 3.0: a state-of-the-art annotation of the Kaposi’s sarcoma-associated herpesvirus transcriptome using cross-platform sequencing
Source: mSystems. 2024 Jan 11;9(2):e01007-23. doi: 10.1128/msystems.01007-23 (PMC10878076; doi:10.1128/msystems.01007-23)
Supplement: Figure S4 — Total transcriptome of KHSV. [file msystems.01007-23-s0004.pdf]

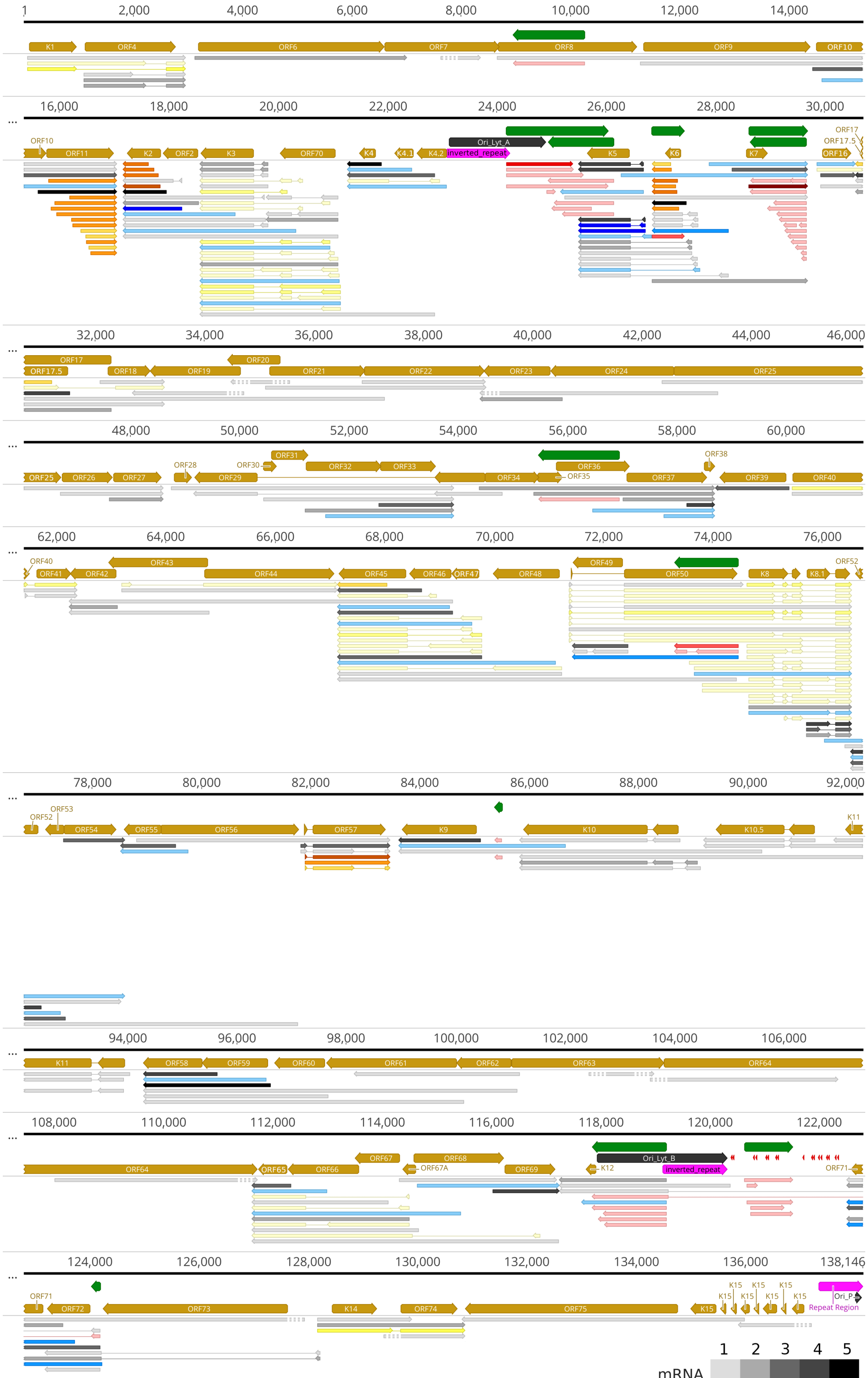

Supplemental Figure 4. The total transcriptome of KHSV with transcript end isoforms and putative embedded genes

Canonical mRNAs, defined as the most abundant RNA isoforms, are represented by black/gray arrows, while the other transcript isoforms are shown as blue arrows. The non-coding transcripts are symbolized with red arrows. Truncated transcripts are indicated by orange arrows. The miRNAs are shown by red arrowheads. Green arrows represent asRNAs. For transcripts where no complete RNA transcripts were detected, the absent portions of the RNA transcripts were denoted with striped lines. The relative abundance of viral transcripts is denoted by varying shades: 1: 1-9 reads, 2: 20-49 reads, 3: 50-199 reads, 4: 200-999 reads, 5: >1000 reads.
