# Supplementary material for: KSHV 3.0: a state-of-the-art annotation of the Kaposi’s sarcoma-associated herpesvirus transcriptome using cross-platform sequencing
Source: mSystems. 2024 Jan 11;9(2):e01007-23. doi: 10.1128/msystems.01007-23 (PMC10878076; doi:10.1128/msystems.01007-23)
Supplement: Figure S5 — Comparison of TIS and TSS distribution along the entire viral genome. [file msystems.01007-23-s0005.pdf]

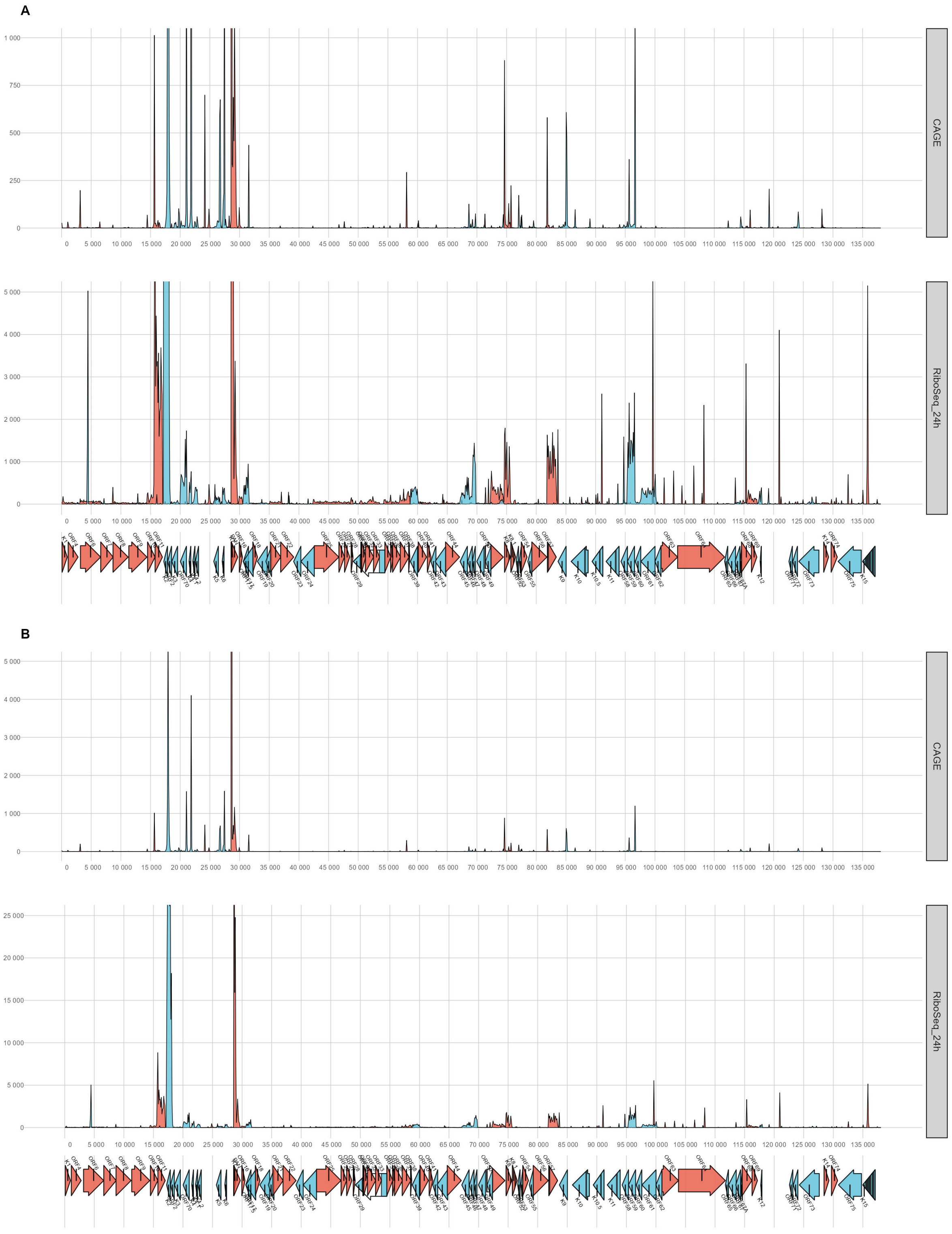

Supplemental Figure 5. Comparison of TIS and TSS distribution along the entire viral genome

Panels (A) and (B) display the TSS distribution from CAGE-Seq and the TIS distribution from RiboSeq, respectively. For each nucleotide a signal strength value was calculated by counting the reads with their 5' ends at that position. These values were subsequently summed within 100-nt windows to produce the displayed distributions. For panel (A), the y-axis is capped at 1000 counts for CAGE-Seq and 5000 counts for RiboSeq. In panel (B), the limits are 1000 counts for CAGE-Seq and 5000 counts for RiboSeq. The distributions are the KSHV genome annotations with ORFs shown beneath. Gene orientations are color-coded: red for the positive strand and blue for the negative strand.
