# Supplementary material for: KSHV 3.0: a state-of-the-art annotation of the Kaposi’s sarcoma-associated herpesvirus transcriptome using cross-platform sequencing
Source: mSystems. 2024 Jan 11;9(2):e01007-23. doi: 10.1128/msystems.01007-23 (PMC10878076; doi:10.1128/msystems.01007-23)
Supplement: Figure S6 — The coding capacity of KSHV. [file msystems.01007-23-s0006.pdf]

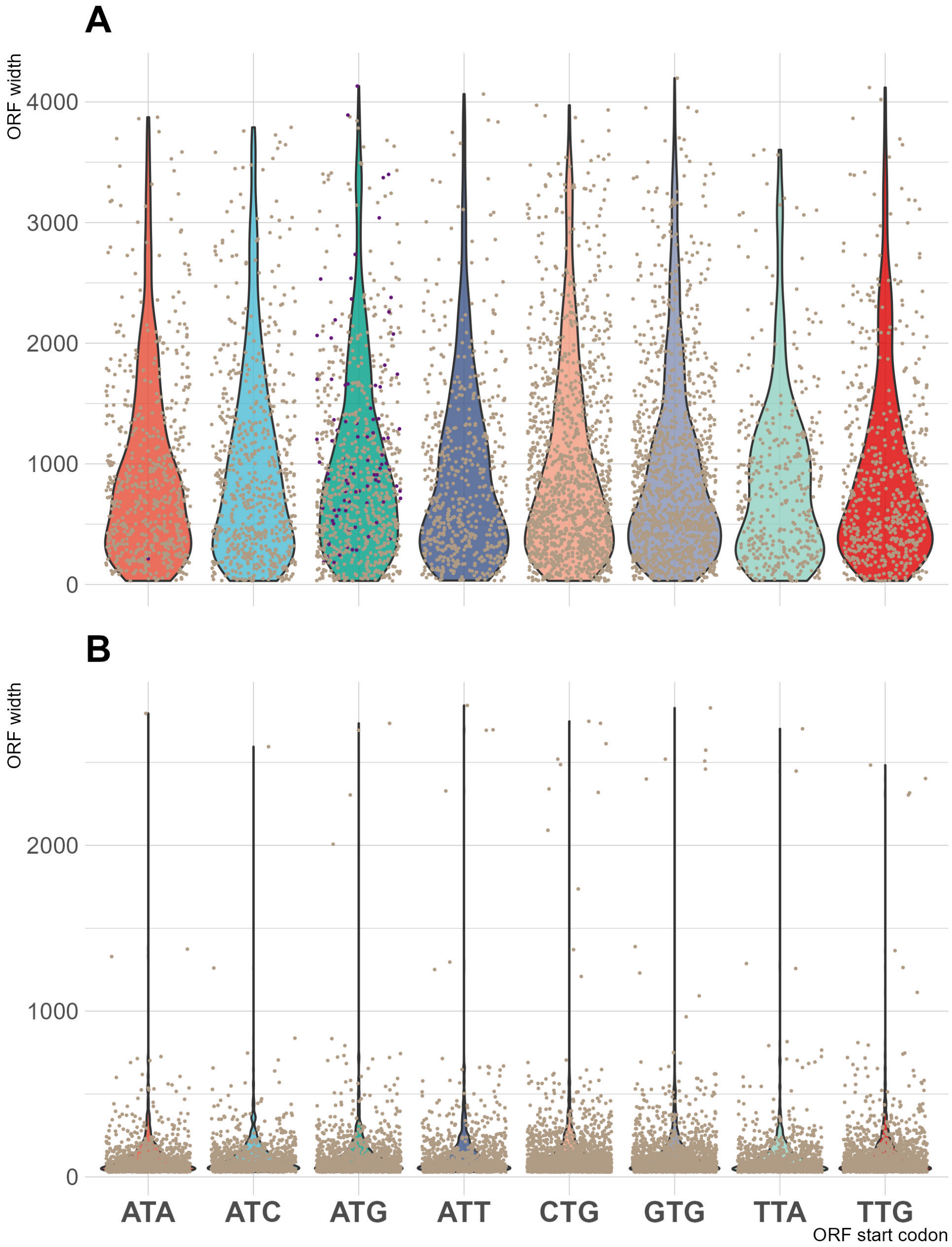

Supplemental Figure 6. The coding capacity of KSHV

The figure shows the widths of all the predicted ORFs in the KSHV genome according to their start codons. Each point represents a single ORF, with the y-axis showing its width (nt) and the x-axis its start codon. Points are colored with blue in the case of canonical ORFs (all of them starting with ATG), otherwise they are colored with light brown. The upper panel (A) shows those ORFs that are coterminal with a canonical ORF, while the lower panel (B) shows those that are not.
