# Supplementary material for: KSHV 3.0: a state-of-the-art annotation of the Kaposi’s sarcoma-associated herpesvirus transcriptome using cross-platform sequencing
Source: mSystems. 2024 Jan 11;9(2):e01007-23. doi: 10.1128/msystems.01007-23 (PMC10878076; doi:10.1128/msystems.01007-23)
Supplement: Figure S7 — Ribosome footprint signals on 5′-truncated transcripts. [file msystems.01007-23-s0007.pdf]

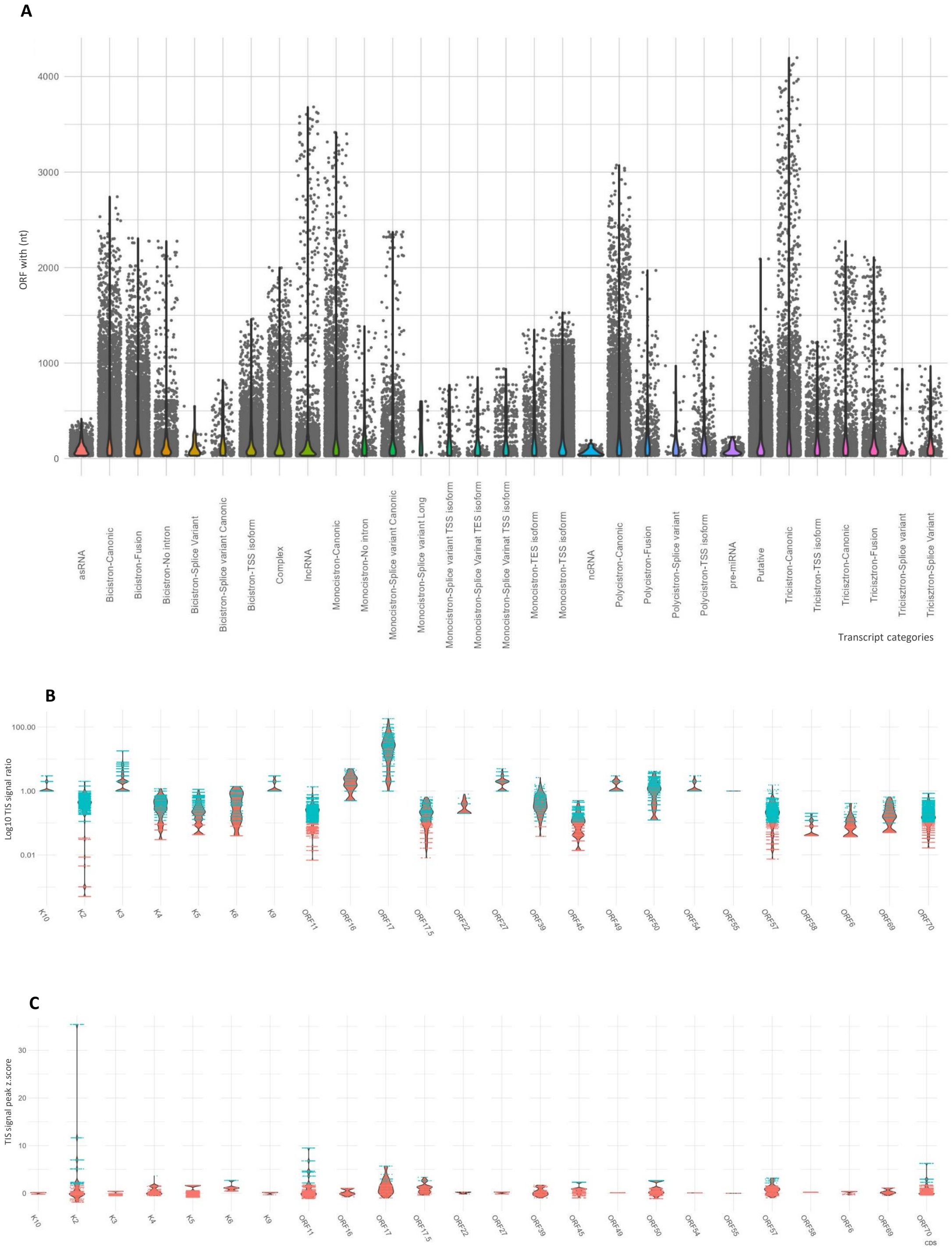

Supplemental Figure 7. Ribosome footprint signals on 5′-truncated transcripts

Panel **(A)** shows the potential coding capacity of the different annotated transcripts according to their types, including mono-, bi-, polycistronic, and 5′ truncated transcripts.

Significant RiboSeq footprint signals on 5′-truncated transcripts in the 24h CHX RiboSeq sample. Panel **(B)** shows the signal strength of the predicted ifORFs encoded by the truncated transcripts. Each point represents a single predicted ORF for each transcript. The x-axis shows the name of the host ORF, while the y-axis shows the ratio of the TIS strength around the predicted ORF, and that of its host ORF. The TIS signal for each in-frame ORF was calculated as the sum of the read counts around +/- 2 nts of its start position. Panel (**C**) shows the result of the significant TIS peak detection on the 5′ truncated transcripts. Each point represents a TIS peak. The x-axis shows the canonical ORFs, while the z-score of each TIS peaks are depicted on the y-axis. Red points represent peaks with insignificant, while blue points represent peaks with significant p-values (significance cutoff = 0.05).
