# Supplementary material for: KSHV 3.0: a state-of-the-art annotation of the Kaposi’s sarcoma-associated herpesvirus transcriptome using cross-platform sequencing
Source: mSystems. 2024 Jan 11;9(2):e01007-23. doi: 10.1128/msystems.01007-23 (PMC10878076; doi:10.1128/msystems.01007-23)
Supplement: Figure S8 — A novel OriLyt-spanning protein-coding transcript. [file msystems.01007-23-s0008.pdf]

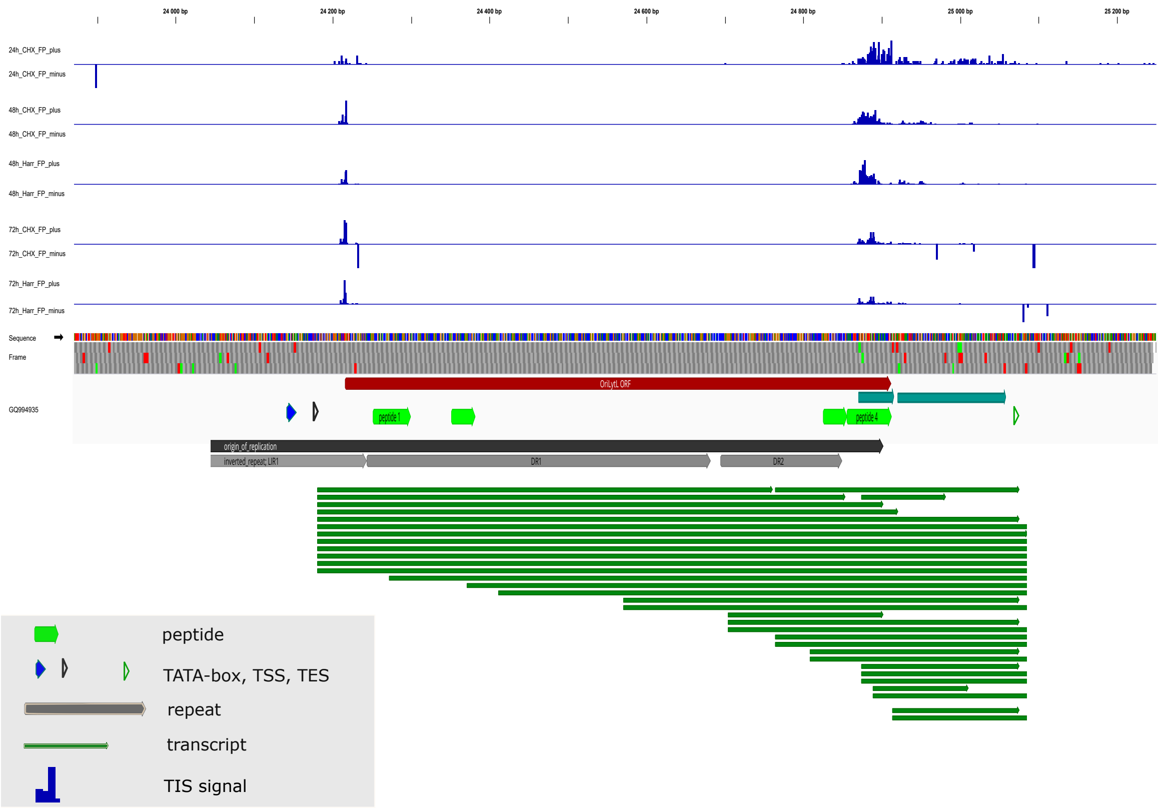

Supplemental figure 8. A novel, OriLyt-spanning, protein coding transcript

There is a group of transcripts overlapping the direct repeats at the intergenic region of OriLyt-L (green). Annotated features, like promoter, TSS and TES are marked as well as footprints of the Ribo-Seq signals obtained from Arias et al., (41) (upper panel). Peptides from Dresang et al. (54) are marked as green arrows.
