# Supplementary material for: KSHV 3.0: a state-of-the-art annotation of the Kaposi’s sarcoma-associated herpesvirus transcriptome using cross-platform sequencing
Source: mSystems. 2024 Jan 11;9(2):e01007-23. doi: 10.1128/msystems.01007-23 (PMC10878076; doi:10.1128/msystems.01007-23)
Supplement: Supplemental Text — Detailed materials and methods. [file msystems.01007-23-s0009.pdf]

KSHV 3.0: A State-of-the-Art Annotation of the Kaposi's Sarcoma-Associated Herpesvirus Transcriptome Using Cross-Platform Sequencing

István Prazsák[#1], Tombácz D[1#], Ádám Fülöp[#1], Torma G[1], Gulyás G[1], Dörmő Á[1], Kakuk B[1], McKenzie Spires L[2], Toth Z[2*], Boldogkői Z[1*]

MATERIALS AND METHODS

RNA isolation

Total RNA was extracted from KSHV-infected cells utilizing the NucleoSpin® RNA kit (Macherey-Nagel). The spin-column method was utilized for the process. In summary, cells were disrupted by adding a buffer solution containing chaotropic ions (provided in the kit). Next, nucleic acids were attached to a silica membrane. To eliminate genomic DNA, DNase I treatment was performed on the samples. Total RNAs were then collected using RNase-free water. The TURBO DNA-free™ Kit was employed to remove any potentially remaining DNA from the samples, which were then preserved at -80°C.

Poly(A) selection

The Lexogen Poly(A) RNA Selection Kit V1.5 was employed to select polyadenylated RNAs from the KSHV total RNA samples. In brief, 10 µL of total RNA (5 µg) was denatured at 60°C for 1 minute and then held at 25°C. The RNA samples were mixed with 10 µL of washed beads (included in the kit), and the mixtures were incubated at 25°C for 20 minutes with agitation at 1,250 rpm. Subsequently, the tubes were placed on a magnetic rack for 5 minutes, and the supernatant was discarded. The beads were then resuspended in Bead Wash Buffer (provided by the Lexogen kit) and incubated at 25°C for 5 minutes with agitation at 1,250 rpm. Afterward, the tubes were moved to the magnetic rack and the supernatant was removed following a 5-minute incubation. This washing step was performed twice. Following the second wash, the beads were resuspended in Nuclease-free water (from the Lexogen kit) and incubated at 70°C for 1 minute. The tubes were then placed on the magnet for 5 minutes, and the supernatant containing the poly(A)+ RNA fraction was transferred to a new tube.

Measurement of nucleic acid quality and quantity

The Qubit 4.0 fluorometer and Qubit Assay Kits (Invitrogen) were employed for measuring RNA concentration. The RNA BR Assay was used to quantify total RNA samples, while the RNA HS Assay was applied for poly(A)+ and ribodepleted RNA fractions. The quality of total RNA samples was evaluated using the Agilent TapeStation 4150 device with RNA ScreenTape and reagents. RIN scores above 9.6 were selected for cDNA production. The Qubit dsDNA HS Assay Kit (Invitrogen) was utilized for quantifying cDNA samples. To analyze the quality of Illumina libraries, the Agilent High Sensitivity D1000 ScreenTape and the Agilent TapeStation 4150 device were employed.

Cap Analysis of Gene Expression (CAGE)

We used CAGE-Seq to examine the TSS distribution patterns across the entire KSHV genome with three biological replicates. The CAGE™ Preparation Kit (DNAFORM, Japan) was employed for this purpose. Following the manufacturer's guidelines, CAGE libraries were prepared from 1 µg of poly(A)-selected RNA. We chose to utilize poly(A)-selected samples for this analysis because our goal was to employ this method to validate the LRS data, which were also produced using the same technique.

Subsequently, the RNA and RT primer (random primer mixture from the CAGE™ Prep Kit) were denatured at 65°C for 5 minutes. The first cDNA strands were synthesized using SuperScript III Reverse Transcriptase (Invitrogen). To enhance the RT enzyme's activity and specificity, a trehalose/sorbitol mixture (CAGE™ Prep Kit) was also added. The reactions were incubated for 30 seconds at 25°C, followed by the RT reaction at 50°C for 60 minutes.

Diol groups in the 5'-end Cap (and ribose at the 3'-end) were oxidized with NaIO4, and Biotin (long arm) hydrazine was attached to it. First, oxidation was performed by adding NaOAc (1M, pH 4.5, CAGE™ Prep Kit) and NaIO4 (250mM, CAGE™ Prep Kit) to the samples and incubating on ice for 45 minutes in the dark. Following this step, 40% glycerol and Tris-HCl (1M, pH 8.5, CAGE™ Prep Kit) were added to the samples. NaOAc (1M, pH 6.0) and Biotin Hydrazine (10 mM, CAGE™ Prep Kit) were combined with the samples, and the oxidized diol residues were biotinylated at 23°C for 2 hours. Afterward, single-strand RNA was digested by applying RNase I (CAGE™ Prep Kit) treatment (37°C for 30 minutes).

Biotinylated, capped RNA samples were mixed and bound (Cap-trapping) to the pretreated Streptavidin beads (pretreatment details below) at 37°C for 30 minutes, followed by incubation on a magnetic rack. The beads were washed twice with Wash Buffer 1, then with Wash Buffer 2, and finally with Wash Buffer 3 (all from the CAGE™ Prep Kit). Subsequently, cDNAs were released from the beads: Releasing Buffer was added to the samples, and they were incubated at 95°C for 5 minutes. After a brief incubation on a magnetic rack, the supernatant (containing the capped cDNAs) was transferred to new tubes.

RNase I buffer (CAGE™ Prep Kit) was added to the tRNA-Streptavidin bead, and they were placed on a magnetic rack. The supernatant was transferred to the tubes containing the cDNAs and stored on ice. The samples were treated with an RNase mixture (RNase H and RNase I, both from the CAGE™ Prep Kit) and incubated at 37°C for 15 minutes. Any remaining RNA was digested with RNase I, with the reaction conducted at 37°C for 30 minutes.

The Streptavidin beads were coated with tRNA (CAGE™ Prep Kit), mixed, and incubated on ice for 30 minutes before being placed on a magnetic stand for 3 minutes. The supernatant was removed, and the beads were washed twice with Wash Buffer 1 (CAGE™ Prep Kit). Lastly, the beads were eluted in Wash Buffer 1, and tRNA was added. The sample volume was reduced using the miVac DUO Centrifugal Concentrator (Genevac), and then single-strand 5′ linkers (with barcodes) were ligated to the samples at 16°C for 16 hours using the DNA ligation mixture (CAGE™ Prep Kit). After a purification step, the miVac DUO was used again to concentrate the samples. The 3′ linker ligation followed, using the DNA ligation mixture and performed at 16°C for 16 hours. (The 5′ and 3′ linkers were preheated at 55°C, and the cDNA samples at 95°C before ligation steps).

Samples were mixed with Shrimp Alkaline Phosphatase (SAP, CAGE™ Prep Kit) to remove the phosphate group of the linkers. The reaction was performed at 37°C for 30 minutes and stopped at 65°C for 15 minutes. Next, the USER enzyme was added to the sample to digest the dUTP from the 3′ linker up strand. The USER treatment was conducted at 37°C for 30 minutes and halted at 95°C for 5 minutes. After this, the barcoded samples were combined and concentrated with miVac DUO.

Finally, the second cDNA strands were synthesized with the 2nd primer, DNA polymerase, buffer, and 10 mM dNTP (all from the CAGE™ Prep Kit). The denaturation step was set to 95°C for 5 minutes, annealing to 55°C for 5 minutes, and elongation to 72°C for 30 minutes. The sample mixture was treated with Exonuclease I enzyme (37°C for 30 minutes). The vacuum concentrator was used to completely dry the sample, which was then dispensed in 10 µl of nuclease-free H2O. The quantity of single-stranded cDNAs was measured using Qubit 2.0 and the Qubit ssDNA HS Assay Kit.

RNAClean XP Beads were used after RT, oxidation, and biotinylation. AmpureXP Beads were used to purify the samples after Cap-trapping and Releasing, RNase I treatment, 5′ and 3′ linker ligation, SAP and USER treatments, 2nd strand cDNA synthesis, and Exonuclease I treatment. Libraries with different barcodes were pooled and loaded to the same flow cells. The libraries were sequenced on a MiSeq instrument with v3 (150 cycles) and v2 (300 cycles) chemistries (Illumina). The Qubit 4.0 and 1X dsDNA High Sensitivity (HS) Assay were used to measure the sample concentration. The library quality was assessed using TapeStation.

Promoter identification

An in-house software, SeqTools was applied for the detection of promoter elements and for the assembly of basic statistics (https://github.com/moldovannorbert/seqtools).

Downstream data analysis and visualization

The downstream data analysis and the preparation of sequencing data for several figures were conducted within the R environment, using GenomicRanges (1), tidygenomics (2), and packages from the tidyverse (3) and a custom R workflow. In brief, the '.bam' files were imported into R with the *ov.from.bam2* function [which employs Rsamtools (https://bioconductor.org/packages/Rsamtools)], coverages were calculated and the 3- and 5-prime ends were summed per genomic position in each sample. **Figures 2 and 4** and **Supplemental Figures 1, 2, 3, 5, 6 and 7** were generated from the prepared data (from either the 3- or 5-prime end distributions), along with the genome annotation, using a custom plotting function (*plot.genome.region*), employing ggplot2 and its *geom_point* or *geom_area* 'geoms' for the distributions and gggenes (https://github.com/wilkox/gggenes) for the annotation. These scripts are available on GitHub at https://github.com/Balays/Rlyeh and the complete R workflow is available as a workflow https://github.com/Balays/KSHV_RNASeq. The scripts can also be used to import other alignments into R and create similar plots from their 3' or 5' distributions on the reference genomes.

The R package ORFik was used to predict ORFs on the KSHV genome; using the *findMapORFs* function on our transcript list. In the case of stop codons, the default was used ("TAA|TAG|TGA"), while in the case of start codons, an extend list was used: "TTA|TTG|CTG|ATT|ATC|ATA|ATG|GTG". Peaks from the TIS data were identified using the pracma package's *findpeaks* function in R. After peak detection, the z-score for the mean TIS signal for each peak was calculated and from them, significant peaks were identified using the p-value distribution and a significance cutoff of 0.05. The peaks were associated to the identified transcripts and the predicted ORFs.

References

1. Aboyoun P, Pages H LM. 2010. GenomicRanges: Representation and manipulation of genomic intervals. R Packag version.

2. Ahlmann-Eltze C. 2019. tidygenomics: Tidy Verbs for Dealing with Genomic Data Frames.

3. Wickham H, Averick M, Bryan J, Chang W, McGowan L, François R, Grolemund G, Hayes A, Henry L, Hester J, Kuhn M, Pedersen T, Miller E, Bache S, Müller K, Ooms J, Robinson D, Seidel D, Spinu V, Takahashi K, Vaughan D, Wilke C, Woo K, Yutani H. 2019. Welcome to the Tidyverse. J Open Source Softw 4.
